# Correlation of pelvic ultrasonography with pubertal development in girls

**DOI:** 10.61622/rbgo/2024AO05

**Published:** 2024-03-15

**Authors:** Francine Zap Bertoncello, Mariane Faccin Beust, Cláudia Mendes Tagliari, Liliane Diefenthaeler Herter, Cristiane Kopacek

**Affiliations:** 1 Universidade Federal de Ciências da Saúde de Porto Alegre Porto Alegre RS Brazil Post Graduation Program for Pediatrics, Universidade Federal de Ciências da Saúde de Porto Alegre, Porto Alegre, RS, Brazil.; 2 Hospital da Criança Santo Antônio Pediatric Gynecology Unit Porto Alegre RS Brazil Pediatric Gynecology Unit, Hospital da Criança Santo Antônio, Porto Alegre, RS, Brazil.; 3 Hospital da Criança Santo Antônio Porto Alegre RS Brazil Radiology Service, Hospital da Criança Santo Antônio, Porto Alegre, RS, Brazil.; 4 Universidade Federal de Ciências da Saúde de Porto Alegre Departament of Gynecology Porto Alegre RS Brazil Departament of Gynecology, Universidade Federal de Ciências da Saúde de Porto Alegre, Porto Alegre, RS, Brazil.; 5 Irmandade Santa Casa de Misericórdia de Porto Alegre Porto Alegre RS Brazil Endocrinology Service, Irmandade Santa Casa de Misericórdia de Porto Alegre, Porto Alegre, RS, Brazil.; 6 Universidade Federal do Rio Grande do Sul Departament of Pediatrics Porto Alegre RS Brazil Departament of Pediatrics, Universidade Federal do Rio Grande do Sul, Porto Alegre, RS, Brazil.

**Keywords:** Puberty, Precocious puberty, Ultrasonography, Color doppler ultrasonography, Uterine artery, Women

## Abstract

**Objectives:**

This study aims to correlate pelvic ultrasound with female puberty and evaluate the usual ultrasound parameters as diagnostic tests for the onset of puberty and, in particular, a less studied parameter: the Doppler evaluation of the uterine arteries.

**Methods:**

Cross-sectional study with girls aged from one to less than eighteen years old, with normal pubertal development, who underwent pelvic ultrasound examination from November 2020 to December 2021. The presence of thelarche was the clinical criterion to distinguish pubescent from non-pubescent girls. The sonographic parameters were evaluated using the ROC curve and the cutoff point defined through the Youden index (J).

**Results:**

60 girls were included in the study. Uterine volume ≥ 2.45mL had a sensitivity of 93%, specificity of 90%, PPV of 90%, NPV of 93% and accuracy of 91% (AUC 0.972) for predicting the onset of puberty. Mean ovarian volume ≥ 1.48mL had a sensitivity of 96%, specificity of 90%, PPV of 90%, NPV of 97% and accuracy of 93% (AUC 0.966). Mean PI ≤ 2.75 had 100% sensitivity, 48% specificity, 62% PPV, 100% NPV and 72% accuracy (AUC 0.756) for predicting the onset of puberty.

**Conclusion:**

Pelvic ultrasound proved to be an excellent tool for female pubertal assessment and uterine and ovarian volume, the best ultrasound parameters for detecting the onset of puberty. The PI of the uterine arteries, in this study, although useful in the pubertal evaluation, showed lower accuracy in relation to the uterine and ovarian volume.

## Introduction

Precocious puberty is defined by the appearance of secondary sexual characteristics before girls reach the age of eight years old and can be classified in the following ways: Central Precocious Puberty (CPP), caused by premature activation of the hypothalamic-pituitary-gonad (HPG) axis, usually of idiopathic cause. Peripheral Precocious Puberty (PPP), caused by an increase in sex steroids, independent of the production of gonadotropin-releasing hormone (GnRH), as in cases of autonomous follicular cyst or estrogen-producing ovarian tumor. There are also, the variants of normal puberty: telarche, pubarche or isolated menarche; which are present in an isolated form, in the absence of axis activation or increased production of sex steroids and that do not increase growth velocity neither bone age. These are probably due to an increase in the sensitivity of the receptors to sex steroids.^(1-4)^

Different diagnoses lead to different therapeutic approaches. The initial assessment includes clinical, laboratory and imaging parameters. In laboratory investigation, luteinizing hormone (LH) measurement is crucial to confirm PPC. Although the clinical condition may suggest PPC, the baseline LH, sometimes, is found in prepubertal values. In cases like these, the GnRH stimulation test or a GnRH analogue should be used, these tests, however, are expensive, invasive, painful and time consuming.^(5,6)^

Pelvic ultrasound, on the other hand, is a simple, non-invasive, available and low-cost test, with an important role in the assessment of precocious puberty. It rules out the presence of ovarian cysts and neoplastic lesions and allows the assessment of whether there is hormonal stimulation in pelvic organs. Several studies have tried to define cutoff points to differentiate prepubertal and pubertal girls, a rather arduous task, as pubertal development is a *continuum* and there is an overlap of what is considered normal and what is considered pathological. Therefore, a new ultrasound parameter has been studied, the Doppler evaluation of the uterine arteries, which could be useful in the evaluation of precocious puberty.^(7,8)^

The purpose of this research is to describe and correlate pubertal changes (Breast Tanner Stages) with the development of the internal genitalia; and evaluate the usual ultrasound parameters and the Doppler study of the uterine artery as diagnostics tests for the onset of puberty.

## Methods

A cross-sectional study carried out between November 2020 and December 2021. Girls aged from one to less than eighteen years old, who were referred to the diagnostic imaging service of *Hospital da Criança Santo Antônio* (HCSA), to undergo pelvic, urinary tract or abdomen ultrasound, were invited to participate in the study.

Exclusion criteria presence of thelarche or pubarche before eight years of age, use of GnRH analogue, current or past six months of hormonal contraceptive use, and presence of severe comorbidities that may interfere with normal pubertal growth and development.

Study participants, together with their guardians, answered a short questionnaire about clinical data. The physical examination was performed on the same day as the ultrasound by two specialists in pediatric gynecology, the breast examination was done with the participants in the supine position.

The measurements of the breast, areola and nipple were obtained with a measuring tape. Pubertal development was classified according to the Breast Tanner Stages.^(9)^ The criterion used to distinguish pubescent from non-pubescent girls was the presence of thelarche. Patients were divided into three groups: prepubertal (Tanner 1), initial puberty (Tanner 2 and 3) and late puberty (Tanner 4 and 5) for further comparison.

Pelvic ultrasound examinations were performed through the abdomen by a pediatric radiologist with experience in pelvic ultrasound in children. Uterine and ovarian volume were calculated according to the formula for prolate ellipse: volume (cm³) = longitudinal diameter (cm) × transverse diameter (cm) × anteroposterior diameter (cm) × 0.5233. After the Doppler signal of the right and left uterine arteries was evaluated and the pulsatility index (PI) calculated, defined as (systolic velocity - diastolic velocity)/mean velocity. The mean PI of both uterine arteries was considered for statistical analyses.

Qualitative variables were calculated using absolute and relative frequencies. Quantitative variables were calculated using the mean and standard deviation or median and interquartile range. The normal distribution of variables was assessed using the Shapiro-Wilk test. For bilateral variables the mean was considered, since the Wilcoxon test showed no difference between the sides. To assess the association between categorical variables, the Pearson's Chi-Squared test was used.

Analysis of Variance (ANOVA) and Kruskal-Wallis tests were used to compare continuous variables with and without normal distribution, respectively, with Bonferroni's post hoc test for multiple analyses. Spearman's correlation test was used for quantitative variables without normal distribution. In order to differentiate prepubescent girls (Tanner 1) from pubescent girls (Tanner 2,3,4 and 5) by means of ultrasound variables and define cutoff points, the receiver operating characteristic (ROC) curve, the area under the curve (AUC) and 95% confidence interval (CI) was used. The cutoff point was defined using the Youden index (J) which is the optimal cut-point when equal weight is given to sensitivity and specificity. Then sensitivity, specificity, positive predictive value (PPV), negative predictive value (NPV) and accuracy were calculated. The significance level used was 5% (p = 0.05) and the analyses using the SPSS statistical software (IBM SPSS Statistics for Windows, Version 25.0. Armonk, NY: IBM Corp.).

An informed consent was applied to the parents or guardians of the children or adolescents who accepted the invitation to participate in the research. The project was submitted and approved by the Research Ethics Committee of the HCSA under number 35997020.9.0000.5683.

## Results

**Figure 1:**
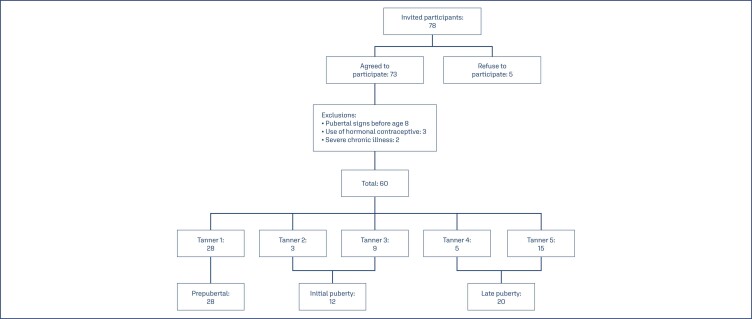
Flowchart of inclusion of study participants

Sixty girls aged between one and seventeen years and six months were included in the study (Figure 1), with a mean of 8.94 years (±4.42). Twenty-eight of them were in the prepubertal group, twelve in the initial puberty group and twenty in the late puberty group.

**Figure 2:**
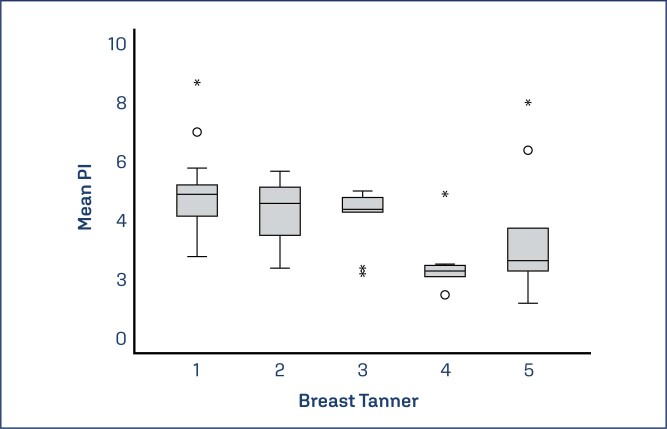
Boxplot of mean IP values according to Tanner Stage

**Figure 3:**
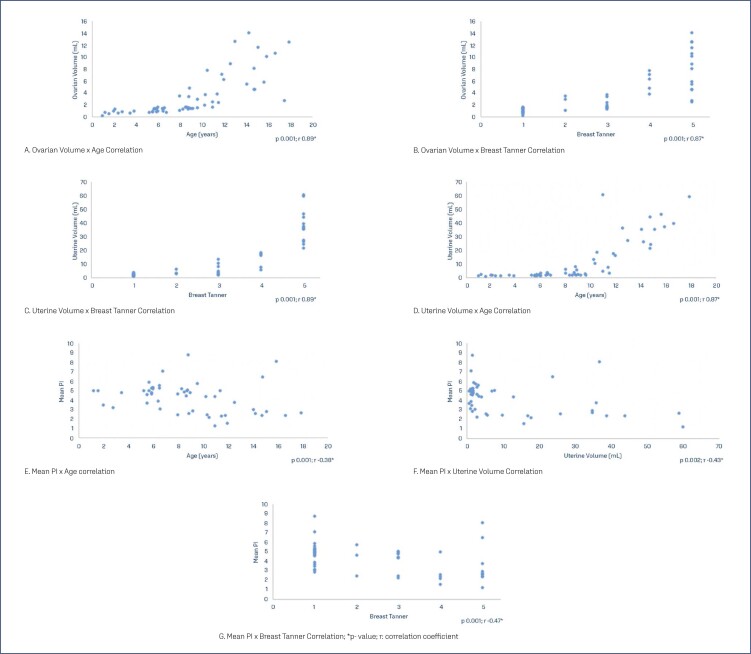
Correlations between Tanner Stages, age and ultrasound variables

Comparing the groups (Table 1), there was a progressive increase in uterine volume, uterine length, ovarian volume and transverse nipple diameter as pubertal development increased. Endometrial thickness was greater in girls in late puberty compared to prepubertal and early pubertal ones, with no difference between prepubertal and initial puberty girls. The number of follicles in late pubertal girls was higher than in prepubertal girls. There was no difference between the PI in the three groups, but when compared the Breast Tanner Stages separately, it was observed that there was a significant difference between Breast Tanner 1 and 4 with p-value of 0.012 (Table 2) (Figures 2 and 3).

**Table 1:** Clinical and ultrasound data of the sample population and comparison between groups

Variables		Mean value	SD %	Prepubertal (n=28) Mean value	SD %	Initial puberty (n=12) Mean value	SD %	Late puberty (n=20) Mean value	SD %	p-value
Age		8.94	4.42	5.23	2.40	9.36	1.16	13.87	2.41	0.001
Ethnicity										0.78
	White	47	78.3	23	82.1	8	66.7	16	80	
	Brown	9	15	3	10.7	3	25	3	15	
	Black	3	5	1	3.6	1	8.3	1	5	
	No information	1	1.7	1	3.6	0	0	0	0	
Menarche										0.01
	Yes	15	25	0	0	1	8.3	14	70	
	No	44	73.3	28	100	11	91.7	5	25	
	No information	1	1.7	0	0	0	0	1	5	
Age of menarche		11.52	1.4			10		11.63	1.38	
Acanthosis nigricans										0.656
	Yes	4	6.7	1	3.6	1	8.3	2	10	
	No	56	93.3	27	96.4	11	91.7	18	90	
	Weight (kg)	35.97	17.97	22.47	10	36.18	7.93	54.75	13.57	0.001
	Weight percentile	65.5	25.8	63.7	29.9	68.5	26.8	66.2	19.2	0.862
	Height (cm)	130.4	24.9	109.2	18.3	137.7	6.9	155.6	8	0.001
	Height percentile	51.91	30.45	51.46	32	60.46	37.13	47.41	23.63	0.507
	BMI (Kg/m^2^)	19.73	4.63	18.02	4.19	18.94	3.55	22.59	4.58	0.002
	Uterine length (cm)	3.6	1.2	3	0.6	3.9	0.8	5.8	1.3	0.018
	Uterine volume (mL)	11.3	15.8	1.6	0.6	4.9	3.6	30.7	15.7	0.001
	Body cervix ratio	1.2	0.4	1	0.1	1.3	0.6	1.5	0.5	0.302
	Endometrium (cm)	0.2	0.3	0	0	0.1	0.1	0.6	0.3	0.029
	Mean PI	4.2	1.6	4.8	1.3	4.1	1.2	3.2	1.9	0.230
	Breast diameter (cm)	3.5	3.9	0.1	0.4	3.4	1.1	8.2	2.5	0.001
	Areola diameter (cm)	2.2	1.2	1.4	0.5	1.9	0.7	3.4	1.1	0.026
	Nipple diameter (cm)	0.4	0.2	0.2	0.1	0.3	0.1	0.6	0.3	0.016
	Mean ovarian volume (mL)	3.3	3.6	1	0.4	2.1	0.9	7.5	3.5	0.001
	Largest follicle diameter (cm)	0.58	0.26	0.45	0.13	0.61	0.22	0.8	0.34	0.095
	Number of follicles	5.42	3.18	3.75	2.12	5.68	2.84	8.54	2.93	0.026

**Table 2:** Mean PI comparison according to Tanner Stage

	Tanner 1*	Tanner 2	Tanner 3	Tanner 4*	Tanner 5	p-value
Mean PI	4.8 SD 1.3	3 SD 4.2	4.1 SD 1.1	2.7 SD 1.3	3.5 SD 2.1	0.012

* Kruskal-Wallis with post hoc Bonferroni test

**Figure 4:**
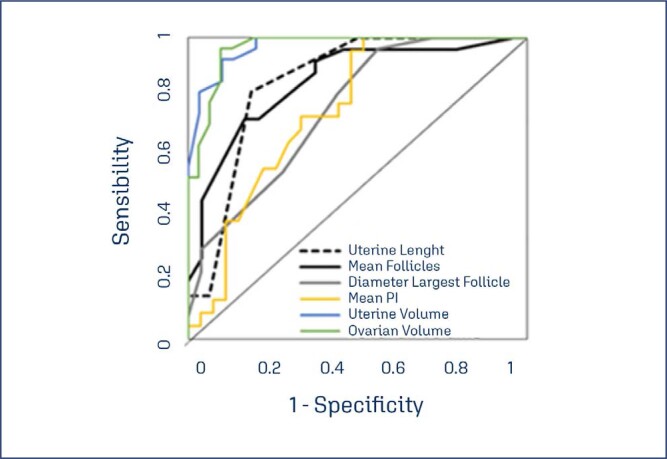
ROC curve of ultrasound diagnostic tests

By using the Spearman test, it was shown that there is a significant correlation between the ultrasound variables with the Breast Tanner and age (Table 3) and (Figure 4). A significant and positive correlation was also found between the Tanner Stages and the transverse diameter of the breast (p 0.001; r 0.95), the transverse diameter of the areola (p 0.001; r 0.79) and the transverse diameter of the nipple (p 0.001; r 0.79). 0.001; r 0.71).

**Table 3:** Correlations between Tanner Stages, age and ultrasound variables

Correlations	Coefficient - r	p-value
Ovarian Volume x Age	0.89	0.001
Ovarian Volume x Age	0.87	0.001
Uterine Volume x Breast Tanner	0.89	0.001
Uterine Volume x Age	0.87	0.001
Mean PI x Age	- 0.38	0.001
Mean PI x Uterine Volume	-0.43	0.002
Mean PI x Breast Tanner	-0.47	0.001

Ultrasound variables were evaluated as diagnostic tests for onset of puberty. Girls in Tanner's 1 were considered prepubescent and the other pubertal patients (Breast Tanner 2,3,4 and 5). Table 4 summarizes the results. Additional cutoffs were presented in supplementary material (Table 5).

**Table 4:** Ultrasound variables and diagnostic tests

Tests		Area	Std error	p-value	CI 95%		Cut-off point	Sensibility %	Specificity %	PPV %	NPV %	Accuracy %
Uterine Lenght		0.854	0.068	0.0001	0.72	0.988	≥ 3.5cm	82	81	89	72	82
Uterine Volume		0.972	0.017	0.0001	0;938	1	≥ 2.45mL	93	90	90	93	91
Mean PI		0.756	0.069	0.002	0.621	0.891	≤ 2.75	100	48	62	100	72
Number of Follicles		0.853	0.054	0.001	0.747	0.96	≥ 3.75	73	83	83	74	78
Largest Follicle Diameter		0.761	0.066	0.001	0.632	0.891	≥ 0.75 cm	96	44	65	92	71
Ovarian Volume		0.966	0.021	0.0001	0.925	1	≥ 1.48mL	96	90	90	97	93
Measurable endometrium								100	70	60	100	79

**Table 5:** Full cut-off points for ultrasound variables

Median PI			
Cutoff	Sensitivity	Specificity	Index
0.2	1	0	0
1.35	1	0.037	0.037
1.8	1	0.074	0.074
2.15	1	0.111	0.111
2.25	1	0.148	0.148
2.35	1	0.259	0.259
2.45	1	0.333	0.333
2.55	1	0.407	0.407
2.65	1	0.444	0.444
2.75	1	0.481	0.481
2.85	0.957	0.481	0.438
2.95	0.957	0.519	0.475
3.05	0.913	0.519	0.432
3.25	0.87	0.519	0.388
3.5	0.826	0.519	0.345
3.65	0.783	0.519	0.301
3.75	0.783	0.556	0.338
4.05	0.739	0.556	0.295
4.35	0.739	0.63	0.369
4.45	0.739	0.667	0.406
4.55	0.696	0.667	0.362
4.65	0.652	0.704	0.356
4.75	0.565	0.741	0.306
4.85	0.565	0.778	0.343
4.95	0.391	0.852	0.243
5.05	0.391	0.889	0.28
5.15	0.304	0.889	0.193
5.25	0.217	0.889	0.106
5.4	0.174	0.889	0.063
5.6	0.13	0.889	0.019
5.75	0.13	0.926	0.056
6.1	0.087	0.926	0.013
6.7	0.087	0.963	0.05
7.5	0.043	0.963	0.006
8.35	0.043	1	0.043
9.7	0	1	0
**Uterine Volume mL**			
**Cutoff**	**Sensitivity**	**Specificity**	**Index**
-0.3	0	1	0
0.75	0.036	1	0.036
0.95	0.143	1	0.143
1.15	0.179	1	0.179
1.25	0.286	1	0.286
1.35	0.321	1	0.321
1.45	0.464	1	0.464
1.55	0.5	1	0.5
1.65	0.571	1	0.571
1.75	0.75	0.967	0.717
1.85	0.821	0.967	0.788
1.95	0.857	0.9	0.757
2.2	0.893	0.9	0.793
2.45	0.929	0.9	0.829
2.75	0.929	0.867	0.795
3.2	0.964	0.8	0.764
3.45	1	0.8	0.8
3.85	1	0.767	0.767
4.8	1	0.733	0.733
5.65	1	0.7	0.7
6.5	1	0.667	0.667
7.45	1	0.633	0.633
8.9	1	0.6	0.6
11.5	1	0.567	0.567
14.5	1	0.533	0.533
16.5	1	0.5	0.5
17.5	1	0.467	0.467
19.5	1	0.433	0.433
22.5	1	0.4	0.4
25	1	0.367	0.367
26.5	1	0.333	0.333
31	1	0.3	0.3
35.5	1	0.233	0.233
36.5	1	0.2	0.2
38	1	0.167	0.167
41.5	1	0.133	0.133
45	1	0.1	0.1
52.5	1	0.067	0.067
59.5	1	0.033	0.033
	1	0	0
**Length uterine cm**			
**Cutoff**	**Sensitivity**	**Specificity**	**Index**
1	0	1	0
2.4	0.143	1	0.143
2.9	0.143	0.938	0.08
3.5	0.821	0.813	0.634
4.25	1	0.5	0.5
4.75	1	0.438	0.438
5.35	1	0.25	0.25
5.85	1	0.188	0.188
7	1	0.063	0.063
9	1	0	0
**Number average of follicles**			
**Cutoff**	**Sensitivity**	**Specificity**	**Index**
0	0	1	0
1.5	0.038	1	0.038
2.25	0.192	1	0.192
2.75	0.269	0.958	0.228
3.25	0.462	0.958	0.42
3.75	0.731	0.833	0.564
4.25	0.731	0.792	0.522
4.75	0.808	0.708	0.516
5.25	0.846	0.667	0.513
5.75	0.885	0.625	0.51
6.25	0.923	0.625	0.548
6.75	0.962	0.542	0.503
7.25	0.962	0.417	0.378
7.75	0.962	0.375	0.337
8.25	0.962	0.333	0.295
9	0.962	0.25	0.212
10.75	0.962	0.208	0.17
12.5	1	0.042	0.042
14	1	0	0
**Diameter of the largest follicle cm**			
**Cutoff**	**Sensitivity**	**Specificity**	**Index**
-0.8	0	1	0
0.25	0.074	1	0.074
0.35	0.222	0.96	0.182
0.45	0.296	0.96	0.256
0.55	0.556	0.72	0.276
0.65	0.815	0.56	0.375
0.75	0.963	0.44	0.403
0.85	1	0.28	0.28
0.95	1	0.24	0.24
1.05	1	0.16	0.16
1.25	1	0.12	0.12
1.45	1	0.08	0.08
1.6	1	0.04	0.04
2.7	1	0	0
**Ovarian Volume mL**			
**Cutoff**	**Sensitivity**	**Specificity**	**Index**
-0.85	0	1	0
0.3	0.036	1	0.036
0.475	0.071	1	0.071
0.525	0.107	1	0.107
0.575	0.143	1	0.143
0.65	0.179	1	0.179
0.725	0.25	1	0.25
0.775	0.357	1	0.357
0.85	0.393	1	0.393
0.95	0.536	1	0.536
1.1	0.536	0.968	0.503
1.2	0.607	0.968	0.575
1.225	0.643	0.968	0.611
1.275	0.714	0.935	0.65
1.325	0.786	0.935	0.721
1.375	0.857	0.903	0.76
1.425	0.893	0.903	0.796
1.475	0.964	0.903	0.868
1.525	0.964	0.871	0.835
1.55	0.964	0.871	0.835
1.7	1	0.806	0.806
2.075	1	0.774	0.774
2.375	1	0.742	0.742
2,525	1	0.71	0.71
2.75	1	0.677	0.677
**Ovarian Volume mL**			
**Cutoff**	**Sensitivity**	**Specificity**	**Index**
3.075	1	0.645	0.645
3.325	1	0.613	0.613
3.525	1	0.581	0.581
3.675	1	0.548	0.548
4.1	1	0.516	0.516
4.6	1	0.452	0.452
5.05	1	0.419	0.419
5.575	1	0.387	0.387
5.975	1	0.355	0.355
6.6	1	0.323	0.323
7.35	1	0.29	0.29
7.85	1	0.258	0.258
8.375	1	0.226	0.226
9.375	1	0.194	0.194
10.25	1	0.161	0.161
11	1	0.129	0.129
11.95	1	0.097	0.097
12.45	1	0.065	0.065
13.25	1	0.032	0.032
15	1	0	0

Figure 4 represents the ROC curve of the diagnostic tests.

## Discussion

This research has confirmed that the pelvic ultrasound study has a great value in the pubertal assessment in girls and despite not being the gold standard in the diagnosis of CPP, it can direct clinical thinking. Although the overlapping of ultrasound measurements between pre-puberty and initial puberty makes it difficult to define exact cutoff points, pelvic ultrasound is capable of diagnosing pubertal activation with satisfactory accuracy.^(10-14)^ The best ultrasound parameters found were ovarian and uterine volumes, which are easy to measure in the examination. The Doppler study of the uterine arteries was inferior to the traditional parameters, not to mention that it requires more training for its execution.

The usefulness of the Doppler study of the uterine arteries in the pubertal evaluation has been little studied so far. Laursen et al.,^(15)^ in 1996 found differences when seeking to assess whether there would be changes in uterine arterial flow during pubertal development. Uterine artery PI was similar in girls in Breast Tanner 1 and 2 and decreased significantly at 3 and 4 Stages, with further increase at Tanner 5.^(15)^

Similar results were found in this research. When evaluating the Breast Tanner Stages individually in relation to the mean IP, a significant difference between Tanner 1 and 4 was found. This confirmed the drop in vascular resistance expressed by the PI in the late period of puberty, not in the initial period (Tanner 2). Since a more significant change in the PI of the uterine arteries that might occur in the late period of puberty may justify the lower usefulness of this test in the diagnosis of pubertal activation.

Once the change in uterine arterial flow was proven, Ziereisen et al.,^(16)^ in 2001 sought to determine the potential contribution of the Doppler assessment of the uterine artery during puberty. They evaluated 61 healthy girls aged two to fifteen years old and found a strong inverse correlation of uterine artery PI with right ovarian volume, uterine transverse diameter and uterine length.^(16)^ Likewise, inverse but weak correlations were found with uterine and ovarian volume in this study, with age and with Tanner Stages, thus reaffirming that PI decreases with pubertal development. The main mechanism proposed for the changes in Doppler flow is the presence of estrogen receptors in the artery wall. Estradiol has been shown to decrease vascular resistance by exerting a direct action on the smooth muscle cells of the middle layer of the uterine artery.^(15-17)^

Golestani et al.,^(18)^ in 2008 evaluated sixty girls divided into three groups: girls without pubertal signs; girls with pubertal signs but no menarche; and girls with pubertal signs and menarche. When comparing the groups, they did not find a significant difference in mean PI. However, the same way this study did, they found a significant difference when analyzing uterine and ovarian volume. The study therefore reinforces that uterine and ovarian volume changes are more prominent at puberty compared to PI.^(18)^

A study carried out by Battaglia et al.,^(8)^ in 2002 was the first to define a cutoff point for PI in the diagnosis of precocious puberty. Twenty-nine girls with telarche and pubarche (Breast and Pubic Hair Tanner 2 or 3), before eight years old were evaluated with a GnRH stimulation test. Afterwards, the patients were divided into two groups: no response to the GnRh test (pre-pubescent n=9) and those with a response (CPP n=20). The CPP group had a lower impedance on the Doppler study with a mean PI of 2.29 ± 0.19 and the prepubertal group had a mean PI of 3.28 ±0.37. Thus, a PI ≤ 2.5 was found, similar to the best cutoff point (2.75) of this study, with sensitivity of 86%, specificity of 100%, PPV of 86%, NPV of 100% and accuracy of 89%. Although this study used the gold standard for the diagnosis of CPP, that is, the GnRh test, it evaluated a small number of patients with precocious puberty, limited to Tanner Stages 2 and 3, and did not include patients with physiological puberty. In a second study, carried out in 2003, Battaglia et al.^(19)^ evaluated 69 girls under 8 years of age with pubertal signs and found similar results. Once again, however, it did not include girls with physiological puberty over 8 years of age.^(19)^

The Italian study by Paesano et al.^(20)^ from 2019, the largest study carried out so far, sought to validate a cutoff point for the IP of the uterine arteries. It evaluated 495 girls referred for suspected alterations in pubertal development. The diagnosis of pubertal activation was made more robustly, as it combined clinical parameters (Breast Tanner), ultrasound (uterine length >3.5 cm) and laboratory parameters (GnRh stimulation test), requiring two of the three criteria to diagnose pubertal activation. Nonetheless, it did include girls in Breast Tanner 2 and 3 in the prepubertal group. Prepubertal girls with PPC or physiological puberty differed significantly in ovarian volume, uterine volume and uterine artery PI measurements. In addition, ultrasound variables were evaluated as diagnostic tests for pubertal activation. It was found that, when combining a PI less than 4.6 with a uterine length greater than 3.5 cm, the accuracy was comparable to the LH peak after stimulation, the gold standard for the diagnosis of pubertal activation.

Thus, the association of PI with uterine length obtained an accuracy and sensitivity of 91%, specificity of 90%, NPV of 88% and PPV of 93%. However, evaluating the PI alone, it was lower than the gold standard and similar to the uterine volume, a simpler measure to be performed in the ultrasound examination, which was not highlighted in the study.^(20)^

In a recent Brazilian study by Cheuiche et al.^(21)^ in which 169 healthy girls were evaluated, as well as in the Italian study by Paesano et al.^(20)^ and unlike this study and the one by Golestani et al.,^(18)^ a significant difference was found in the PI of the uterine arteries between pre and post-pubescent girls.^(21)^ This is probably due to the smaller number of the samples in this study and the one carried out by Golestani et al.^(18)^ Nevertheless, in all studies, the ovarian and uterine volume parameters were significantly different between pre and post-pubertal girls, even with a smaller sample, reinforcing that the variation in puberty of these parameters is more pronounced and easier to measure.

The study by Cheuiche et al.,^(21)^ in which the criterion for defining puberty was the Breast Tanner, as it was in our study, found positive results for the ultrasound variables with similar accuracy between them. The highest accuracy found was the uterine volume (80%), followed by the uterine length and mean PI of the uterine arteries with 79% and the right ovarian volume with 78%. The best cutoff point for PI was 5.05, similar to the study by Paesano et al.^(20)^ Chart 1 summarizes the main findings of the ultrasound diagnostic tests.

**Chart 1:** Cutoff points and accuracy of ultrasound diagnostic tests in different studies

Study	Uterine Volume (mL)	Ovarian Volume (mL)	Uterine Length (cm)	Pulsatility Index	Endometrial Echo
Battaglia et al. (2002).^(8)^ n=29	4 (76%)	*	*	≤ 2.5 (89%)	Presence (69%)
Battaglia et al. (2003).^(19)^ n= 69	4 (93%)	*	*	≤ 2.5 (98.5%)	Presence (91%)
Paesano et al. (2019).^(20)^ n=495	2.48 (89%)	1.25 (75%)	3.5 (86%)	≤4.6 (87%)	*
Cheuiche et al. (2022).^(21)^ n= 169	3.75 (80%)	2.15 (78%)	3.55 (79%)	≤ 5.05 (79%)	1.35mm (76%)
Bertoncello et al. (2024). n= 60	2.45 (91%)	1.48 (93%)	3.5 (82%)	≤ 2.75 (72%)	Presence (79%)

* Not evaluated

Reasons for different findings and cutoff points are possibly due to the heterogeneity of participants in different studies, to ethnic differences, to the evolution of the ultrasound equipment quality and the fact that this test is examiner-dependent, as well as to the difference in the method that defines pubertal activation.

In this study, the most important limitation was a small sample of patients, which cannot represent the population and invalidate the results Diagnostic test studies should use the gold standard for comparison, which in this case would be comparison with the GnRH test. However, we did not have this test available in this study and we only used clinical criteria. Consequently, prepubertal girls with isolated thelarche may have been included due to increased sensitivity to estrogens or even the production of estrone in the adipose tissue of overweight and obese girls - the inclusion of girls only in normal puberty decreased this risk. On the other hand, the robustness of the ultrasound data includes the evaluation made by the same experienced radiologist, and in the same ultrasound device, as well as the collection of clinical data by two physicians with equivalent training.

Another interesting data from this study refers to the positive correlation between Breast Tanner and the transverse diameter of the areola and nipple. Little attention has been paid to the maturation of the areola and nipple during puberty, but the clinical evaluation of these structures can be very useful when in doubt about classifying Breast Tanner Stages. In obese patients, it may be difficult to differentiate lipomastia from thelarche. At other times, in girls with small breasts, it may be difficult to differentiate between Breast Tanner 3 and 5.

A Turkish cross-sectional study evaluated 498 girls with normal puberty between the ages of 8 and 17 years-old and performed measurements of the nipple and areola. The study found that nipple and areola measurements were significantly correlated with Tanner. There was a difference between all stages, with a gradual increase in measurements, except between stages 4 and 5.^(22)^ Future studies, with larger samples, may even define cutoff points for distinguishing Tanner stages. In this study, nipple diameter greater than 5mm or areolar diameter greater than 2 cm were compatible with Tanner advanced stages of 4 and 5.

## Conclusion

This study demonstrated that pelvic ultrasound is of great value in female pubertal assessment. Uterine volume greater than 2.45mL and ovarian volume greater than 1.48mL proved to be the best ultrasound parameters to determine the onset of puberty. The uterine artery pulsatility index, in this study, despite being useful in pubertal assessment, is worse when compared with both uterine volume and ovarian volume. Furthermore, it was observed that nipple diameter greater than 5mm or areolar diameter greater than 2cm were compatible with advanced stages of Tanner (stage 4 or 5).
